# Elevated levels of CCR6^+^ T helper 22 cells correlate with skin and renal impairment in systemic lupus erythematosus

**DOI:** 10.1038/s41598-017-13344-w

**Published:** 2017-10-11

**Authors:** Wei Zhong, Yanfang Jiang, Hongshuang Ma, Jiang Wu, Zhenyu Jiang, Ling Zhao

**Affiliations:** 1 Department of Rheumatology, First Hospital, Jilin University, Changchun, 130021 China; 2 Key Laboratory of Zoonosis Research, Ministry of Education, First Hospital, Jilin University, Changchun, 130021 China; 3 Jiangsu Co-innovation Center for Prevention and Control of Important Animal Infectious Diseases and Zoonoses, Yangzhou, 225009 China; 4 Department of Central Laboratory, First Hospital, Jilin University, Changchun, 130021 China; 5 College of Electrical Engineering and Instrumentation, Jilin University, Changchun, 130021 China

## Abstract

Systemic lupus erythematosus (SLE) is an autoimmune disease with a variety of pathological features. Our study investigated the potential role of CCR6^+^ T cells in organ impairment of SLE patients. We analyzed CCR6^+/−^ T cell subset populations and compared the concentrations of IL-22, IFN-γ, TNF-α, and IL-17A cytokines in 67 patients with newly diagnosed SLE and 26 healthy controls. We found that SLE patients had elevated percentages of CCR6^+^ T, CCR6^+^ Th22, Th17, Th17.1, and CCR6^−^ Th2 cell subsets, along with increased concentrations of IL-22, IFN-γ, TNF-α, and IL-17 cytokines. Higher levels of CCR6^+^ T and CCR6^+^ Th22 cells, along with plasma IL-22 were observed in SLE patients with sole skin and/or renal impairment. The percentage of Th22 cells also correlated with Revised Cutaneous Lupus Erythematosus Disease Area and Severity Index (RCLASI) and IgG levels, and inversely correlated with C3 levels in SLE patients with sole skin impairment. SLE patients with sole renal impairment showed a correlation between the percentage of Th22 cells and ESR levels. Our data indicated that CCR6^+^ Th22 cells may contribute to the pathogenesis of new onset SLE patients with skin or renal impairment, and CCR6 may, thus, be a possible therapeutic target for SLE treatment.

## Introduction

Systemic lupus erythematosus (SLE) is an autoimmune disease which impairs the function of various organs, including kidneys, skin, central nervous system and joints^1^. Specifically, CD4^+^ T cells, which normally regulate the beginning and persistence of autoimmunity, have been shown to be involved in the evolution of lupus^1,2^. Despite activated T cells infiltrating the affected skin and kidney tissue^3–5^, their direct role in organ impairment remains unknown.

In SLE, T cells usually show abnormal localization and induction of inflammation by expressing chemokine receptors and abnormal cytokine secretion^5^. Importantly, CC chemokine receptor (CCR) 4 expression has been noted on memory T cells, which presumably helps these cells to traffic into peripheral tissues^6^. In addition, memory T cells, along with B cells and dendritic cells (DCs) have been suggested to express CCR6^7^, which are involved in the recruitment of pathogenic T cells in psoriasis^8^, rheumatoid arthritis^9^, and experimental autoimmune encephalitis^10^. Another chemokine receptor, CXCR3, has also been shown to be preferentially expressed by Th1 cells^11^. Furthermore, the expression of CCR10 on the surface of circulating skin-homing cutaneous lymphocyte-associated antigen T cells contributes to T cell-mediated skin inflammation through CCL27-CCR10 interaction^12^.

Chemokine receptors characterize various subsets of memory Th cells with different effector functions and migratory ability^13^. Due to heterogeneity in their expression, CCR6^+^ Th cell are typically distinguished into several subpopulations, such as IL-17A (also commonly called IL-17)^14^ or IL-22 producing CCR6^+^ T cells. CCR6^+^ cells with Th17 characteristics display CCR4^+^CCR10^−^CXCR3^−^ phenotype^15–17^, while those with Th22 characteristics have a CCR4^+^CCR10^+^ phenotype^16,18^. However, Th17.1 cells, with a CCR6^+^CCR4^−^CXCR3^+^ phenotype, produce both IL-17 and IFN-γ, which were previously thought to be mutually exclusive functional characteristics^19^. Similarly, IL-9-producing Th9 cells are characterized with CCR6^+^CCR4^−^ phenotype^20^. In addition, like CCR6^−^ Th cells, IFN-γ producing Th1 cells also display a CCR6^−^CCR4^−^CCR10^−^CXCR3^+^ phenotype^11,16^, while IL-4, IL-5 and IL-13 producing Th2 cells have a CCR6^−^CCR4^+^CXCR3^−^ phenotype^21^.

Interestingly, CCR6^+^ Th cells have recently been confirmed to play a pro-inflammatory role in autoimmune diseases^22,23^. Th17 cells expressing CCR6 appeared to be more pathogenic and accelerate organ impairment after renal injury^24^ and arthritis^25^ in various animal models. In addition, a genetic association has also been reported between CCR6 gene polymorphisms and susceptibility to lupus nephritis (LN)^26^. However, there have been few studies highlighting the relationship between CCR6^+^ Th cell sub-populations and SLE, especially in patients with organ impairment.

Thus, in our study, we aimed to determine the frequency of circulating CCR6^+^/CCR6^−^ Th cells by flow cytometry in 67 new onset SLE patients and 26 age- and gender-matched healthy controls (HCs). In addition we also examined levels of IL-22, IL-17, TNF-α, and IFN-γ cytokines in parallel, and further assessed the expression correlation of these T cell subsets and cytokines with clinical parameters and severity index of SLE patients with varying organ impairment.

## Result

### Comparison of demographic and clinical characteristics of SLE patients

The comparison of 67 onset SLE patients and 26 matched HCs showed no significant difference in terms of age and gender. The levels of C-reactive protein(CRP) and white blood cell counts between SLE patients and HCs also demonstrated no difference, as shown in Table 1. However, SLE patients displayed significantly higher levels of IgG and erythrocyte sedimentation rate (ESR), while levels of complement factor (C)3, C4 were lower, as compared to HCs. In addition, we also observed varied SLE Disease Activity Index (SLEDAI) score among SLE patients, and some patients were positive for anti- double stranded(ds)DNA and anti-Smith(Sm) antibodies.

**Table 1:** Clinical and laboratory characteristics.

Parameters	SLE patients	HCs
	(n = 67)	(n = 26)
Age (years)	29 (17–62)	29 (17–65)
Gender: female/male	56/11	22/4
Disease duration	67 (100%)^†^	ND
SLEDAI	18 (2–39)	ND
Positive anti-dsDNA	34 (50.75%)	ND
Positive anti-Sm	29 (43.28%)	ND
Positive antinuclear antibodies	57 (85.07%)	ND
ESR (mm/h)	50 (3–118)*	3 (0–5)
CRP (mg/L)	6.83 (0.43–193)	7.8 (0–15)
C3 (IU/mL)	0.51 (0.05–1.62)*	1.13 (0.9–1.8)
C4 (U/mL)	0.08 (0.00–0.5)*	0.27 (0.1–0.4)
IgG (g/L)	14.50 (4.38–36.70)*	11.00 (7.50–15.50)
WBC (10^9^/L)	5.08 (4.12–9.78)	6.77 (4.02–9.88)

Note: Data shown are median (range) or number of cases. SLE, systemic lupus erythematosus; HCs, healthy controls; ND, undetectable; SLEDAI, SLE disease activity index; ESR, Erythrocyte sedimentation rate; CRP, C-reactive protein; IgG, Immunoglobulin G; WBC, White blood cell counts. Normal values: ESR: 0–15 mm/h, CRP: 0–3 mg/L, C3: 0.9–1.8 units/mL, C4: 0.1–0.4 units/mL, IgG: 7.00–16.00 g/L, WBC: 3.50–9.50 × 109/L. *P < 0.05 versus HCs. ^†^All of the patients had clinical symptoms for less than three months.

### SLE patients revealed increased frequency of CCR6^+^, CCR4^+^ and CCR10^+^ Th cells

**Figure 1:**
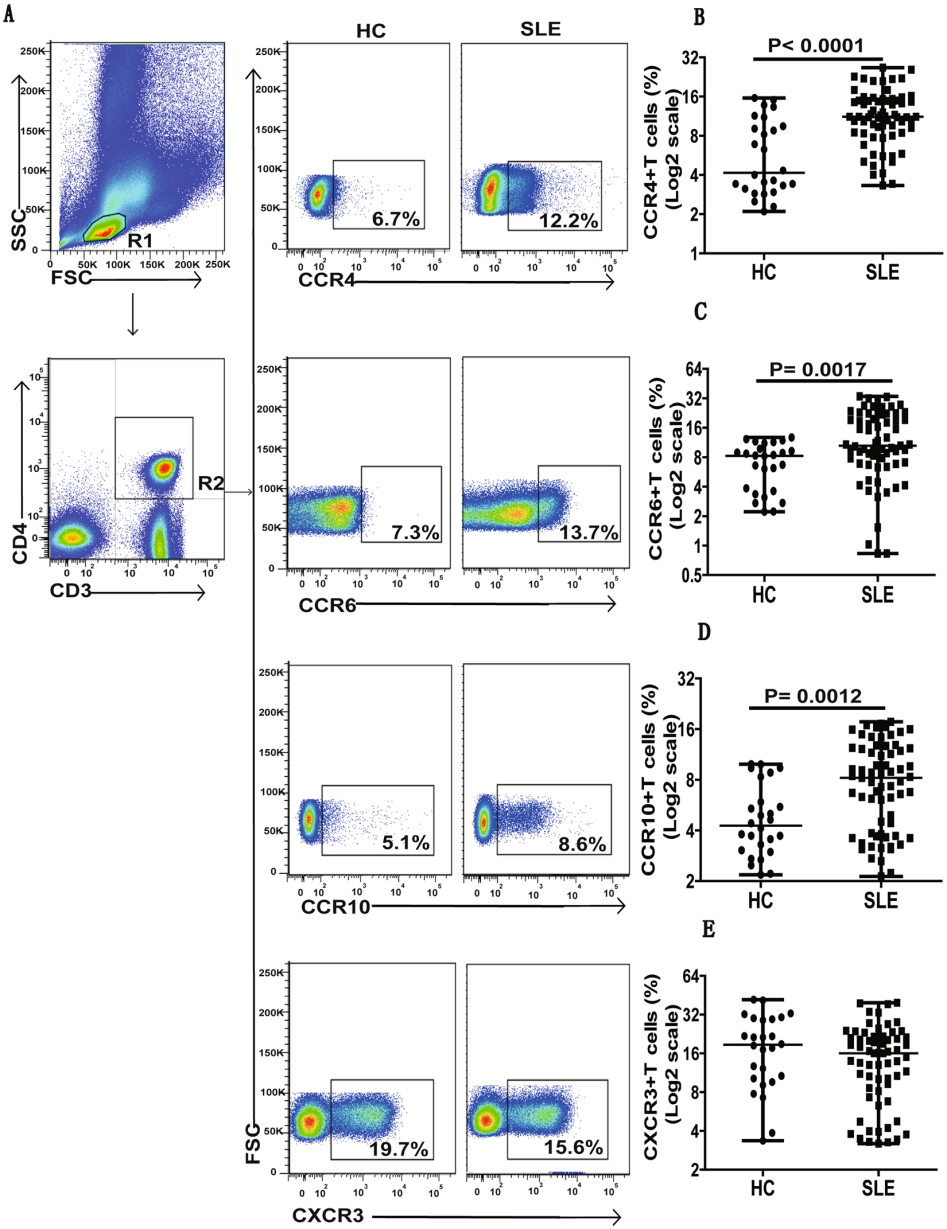
Assessment of circulating CCR4^±^, CCR6^±^ CCR10^±^ and CXCR3^+^ T cells in SLE patients. PBMCs collected from new onset active SLE patients (n = 67) at baseline and HCs (n = 26) were analyzed by flow cytometry for the percentage of different Th cell subsets, including CCR4^+^, CCR6^+^, CCR10^+^ and CXCR3^+^ T cells. Panel (**A**) depicted flow cytometry analysis strategy, while panel (**B–E**) represented quantitative analysis. All dot plots in the data represented mean percentage of Th cells from individual subjects. Differences between groups were analyzed using Mann-Whitney U test. P values < 0.05 represented statistical significance.

It has been previously demonstrated that different chemokine receptors, such as CCR4, CCR6, CCR10 and CXCR3, have varied expression on the surface of Th cell sub-populations^15,16,22^. Our receptor expression analysis indicated significantly increased frequencies of CCR6^+^, CCR4^+^ and CCR10^+^ Th cells in SLE patients, in comparison to HCs. It is noteworthy that we did not observe any significant differences in Th cell sub-population expressing CXCR3 (Fig. 1).

### SLE patients demonstrated elevated proportions of CCR6^+^ Th22, Th17, Th17.1 and CCR6^−^ Th2 cell subsets

**Figure 2:**
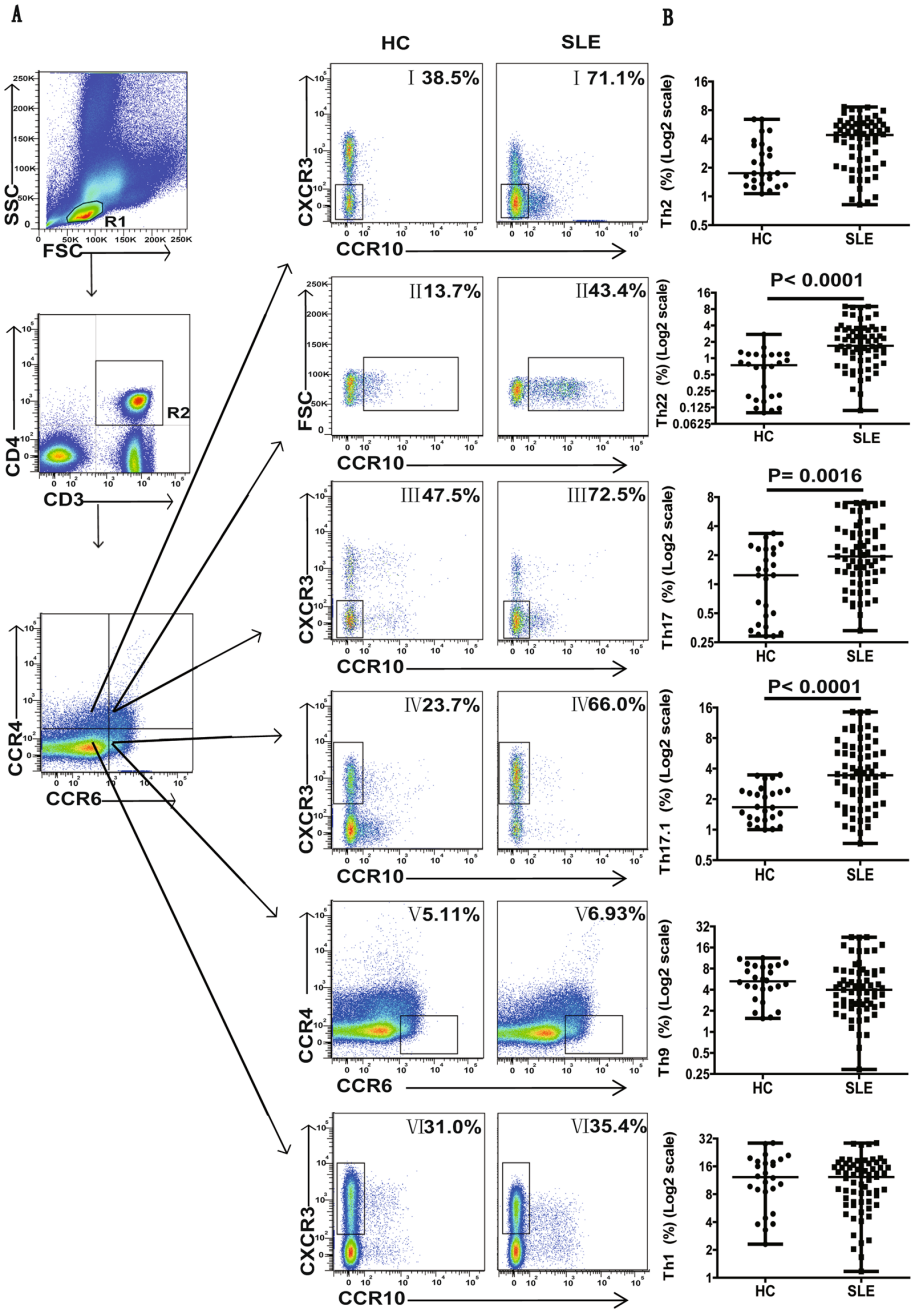
Assessing the percentages of circulating T cell subsets (Th22, Th17, Th17.1, Th2, Th9 and Th1), and the plasma levels of IL-22, IL-17, IFN-γ and TNF-α in SLE patients. PBMCs collected from new onset SLE patients (n = 67) at baseline and HCs (n = 26) were analyzed by flow cytometry to assess the percentage of different Th subsets. Panel (**A**) showed the gating strategy for detecting different Th cell subsets. R1, lymphocytes; R2, CD3^+^CD4^+^ T cells; I, Th2 cells (CCR6^−^CCR4^+^CCR10^−^CXCR3^−^ phenotype); II, Th22 cells (CCR6^+^CCR4^+^CCR10^+^ phenotype); III, Th17 cells (CCR6^+^CCR4^+^CXCR3^-^CCR10^−^ phenotype); IV, Th17.1 cells (CCR6^+^CCR4^−^CCR10^−^CXCR3^+^ phenotype); V, Th9 cells (CCR6^+^CCR4^−^ phenotype); VI, Th1 cells (CCR6^−^CCR4^−^CCR10^−^CXCR3^+^ phenotype). Panel (**B**) represented quantitative analysis of these different cell subsets. All dot plots in each data represented mean percentage of Th cells from individual subjects. Differences between groups were analyzed using Mann-Whitney U test. P values < 0.05 represented statistical significance.

Next, different CD3^+^CD4^+^ T cell subsets (Th22, Th17, Th17.1, Th9, Th2 or Th1) were assessed by using a gating strategy based on chemokine receptor expression (Fig. 2A)^18^. The following chemokine receptor expression profile indicated specific subsets; CCR6^+^CCR4^+^CCR10^+^ phenotype represented Th22 cells, while CCR6^+^CCR4^+^CXCR3^−^CCR10^−^phenotype confirmed Th17 cells. Similarly, CCR6^+^CCR4^−^CCR10^-^CXCR3^+^ phenotype represented Th17.1 cells, and CCR6^+^CCR4^−^ phenotype indicated Th9 subset. Further comparison of these different subsets between SLE patient and HCs, indicated significantly higher frequencies of CCR6^+^ Th subsets (including Th22, Th17 and Th17.1) in SLE patients. However, we did not observe any significant difference in Th9 cells distribution between SLE patients and HCs (Fig. 2B). In addition, we also analyzed CCR6^−^ Th cell subpopulations representing either Th2 (CCR6^−^CCR4^+^ CCR10^−^CXCR3^−^) or Th1 (CCR6^−^CCR4^−^CCR10^−^CXCR3^+^) profiles in SLE patients and HCs. Our results indicated that the proportion of Th2 cells was higher in SLE patients than HCs (Fig. 2B), while no significant difference was observed in Th1 cell subpopulations between these groups (Fig. 2B).

### Positive correlation between CCR6^+^ Th22 cells and IL-22 plasma concentration in SLE patients

**Figure 3:**
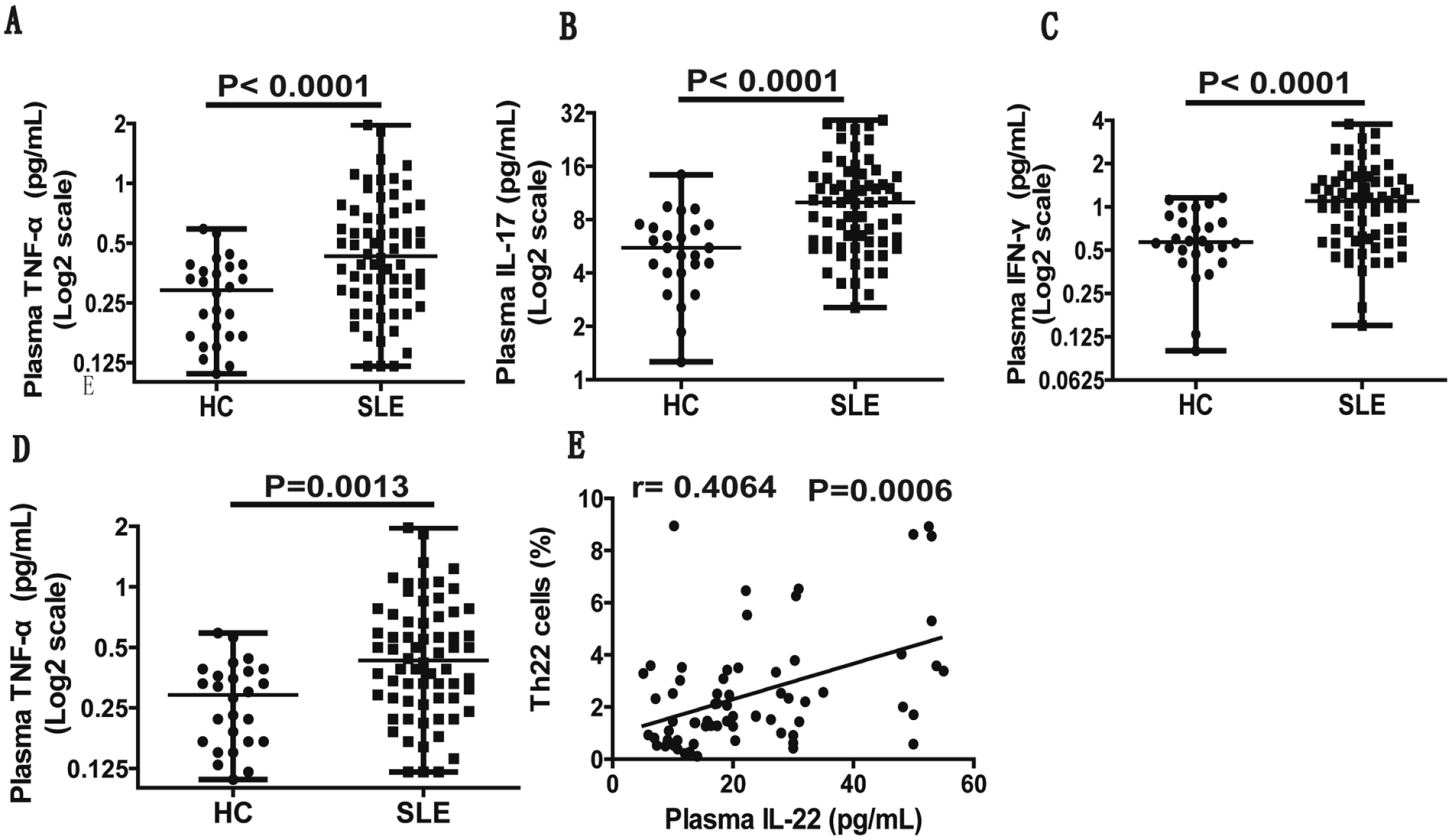
Analysis of the plasma levels of IL-22, IL-17, IFN-γ and TNF-α in SLE patients. Plasma collected from new onset active SLE patients (n = 67) at baseline and HCs (n = 26) was analyzed and compared by ELISA assay for the levels of cytokines IL-22 (panel A), IL-17 (panel B), IFN-γ (panel C) and TNF-α (panel D). Panel (E), represented the correlation between IL-22 plasma level and the percentage of circulating Th22 cells in SLE patients. Differences between groups were analyzed using Mann-Whitney U test. Correlation analysis was performed using Spearman’s rank correlation test. P values < 0.05 represented statistical significance.

In addition, we also determined the concentrations of plasma IL-22, IL-17, IFN-γ, and TNF-α in all the individual subjects, and observed that levels of plasma IL-22 (Fig. 3A), IL-17 (Fig. 3B), IFN-γ (Fig. 3C), and TNF-α (Fig. 3D) were notably elevated in SLE patients in comparison to HCs. We further noted that elevated levels of plasma IL-22 in onset SLE patients positively correlated with Th22 cell population (P = 0.0006 r = 0.4064; Fig. 3E). In contrast, we did not observe a similar correlation of plasma IL-22 concentration with frequencies of other Th cell subsets in SLE patients. Similarly, we also did not find any significant correlation between plasma IFN-γ levels and the frequencies of Th1 or Th17.1 cells, or between plasma IL-17 levels and the frequencies of Th17 or Th17.1 cells, or plasma TNF-α level and the frequencies of different Th cell subsets in SLE patients.

### Correlation of Th cell subsets with clinic indicators in onset SLE patients

**Figure 4:**
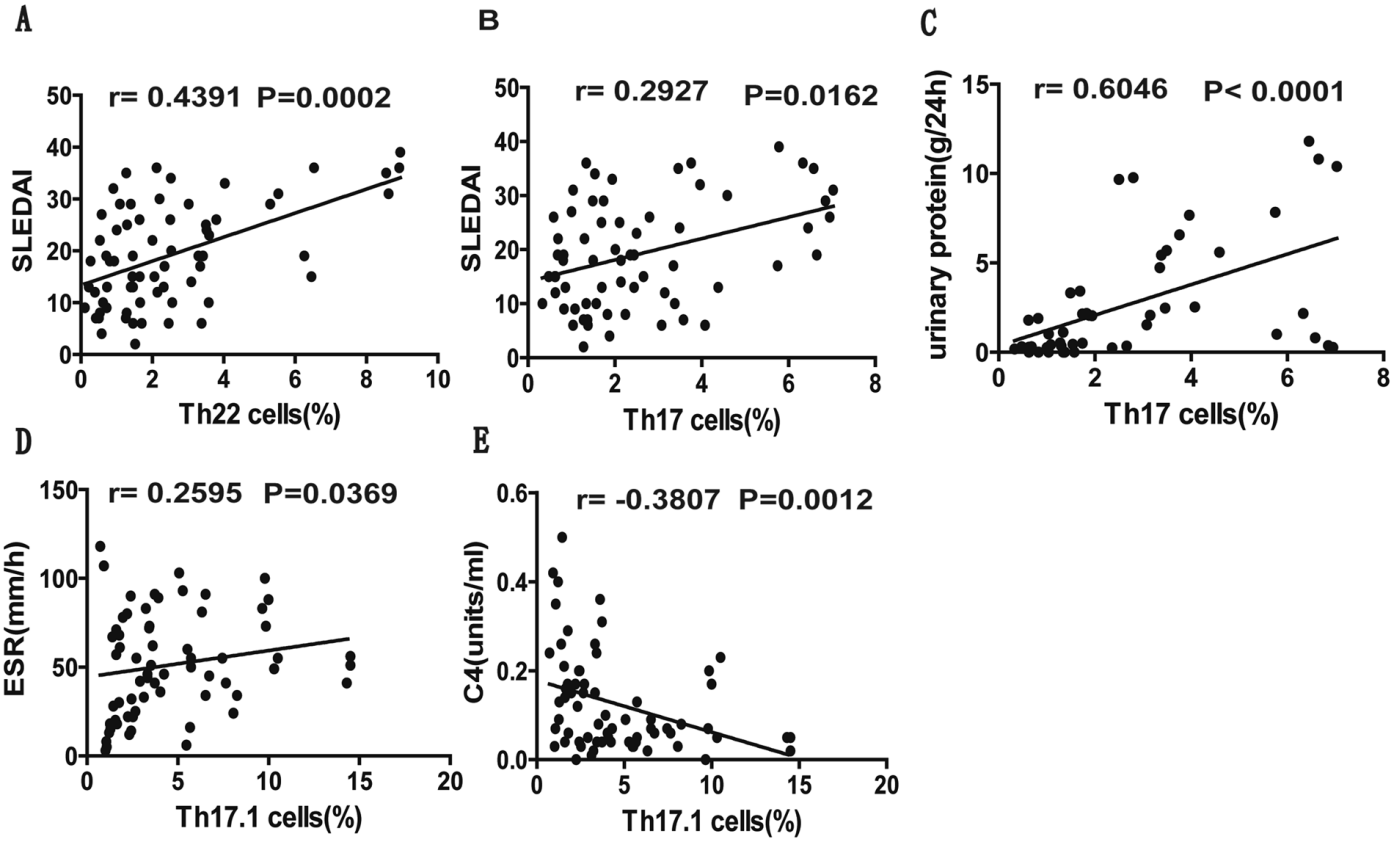
Correlation analysis of different Th cell subsets with SLE clinical parameters and SLEDAI score. Panel (**A**) and (**B**) represented the correlation between SLEDAI score and the percentage of circulating Th22 and Th17 cells, respectively. Panel (**C**) represented the correlation between levels of urinary protein and the percentage of circulating Th17 cells. Panel (**D**) represented the correlation between ESR levels and the percentage of circulating Th17.1 cells. Panel (**E**) represents the correlation between the levels of C4 protein and the percentage of total circulating Th17.1 cells. All analyses were performed using Spearman’s rank correlation test. P values < 0.05 represented statistical significance.

We also examined the potential correlation between Th cell subsets and disease severity in new onset SLE patients. Our data revealed a positive correlation between the percentage of Th22 cells and SLEDAI score in SLE patients (P = 0.0002 r = 0.4391; Fig. 4A). Similarly, the percentage of Th17 cells also showed positive correlation with SLEDAI score (P = 0.0162 r = 0.2927; Fig. 4B) and levels of urinary protein (P < 0.0001 r = 0.6046; Fig. 4C). In addition, Th17.1 cell percentage correlated positively with ESR levels (P = 0.0369 r = 0.2595; Fig. 4D), but inversely correlated with C4 protein (P = 0.0012 r = −0.3871; Fig. 4E). However, no significant correlations were observed between the frequencies of other Th cell subsets with SLEDAI score, and levels of ESR, plasma CRP, Ig, C3, and C4 in SLE patients (data not shown).

### SLE patients with skin and renal impairment showed enhanced CCR6^+^ T and Th22 cell populations

**Figure 5:**
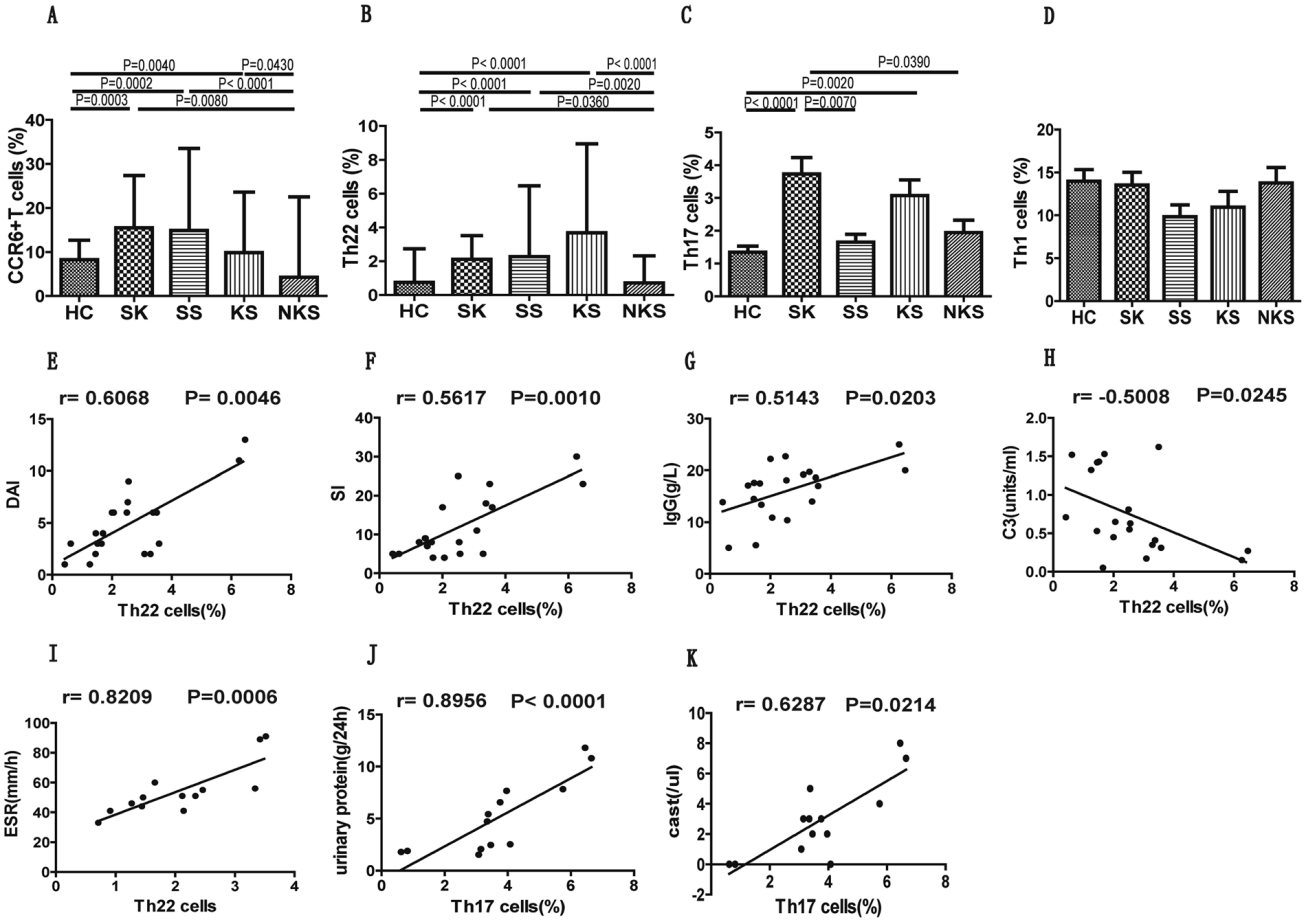
Comparison of different T cell subsets in SLE patients categorized based on organ impairment, and correlation analysis of Th22 and Th17 cells with different clinical parameters of SLE patients with either sole lupus skin disease or sole LN. The percentage of circulating CCR6^+^ T cells (panel **A**), Th22 (panel **B**), Th17 (panel **C**) and Th1 (panel **D**) were analyzed in healthy control and SLE patients from different groups. SK group represented SLE patients with sole renal impairment (n = 13); SS group included SLE patients with sole skin impairment (n = 20); KS group represented SLE patients with both kidney and skin impairment (n = 18), and NKS group represented SLE patients with no renal and skin impairment (n = 16). Differences between groups were analyzed using Kruskal-Wallis ANOVA followed by Dunn-Bonferroni post hoc method. The percentage of circulating Th22 and Th17 cells were analyzed for their correlation with various clinical parameters, using Spearman’s rank correlation test. Panels (**E**–**H**) represented correlation of Th22 cells with DAI (disease area index), SI (severity index), IgG levels, C3 protein, respectively in SLE patients with sole lupus skin disease. Panel (**I**) represented Th22 cells correlation with ESR level, while panel (**J**,**K**) represented Th17 cells correlation with urinary protein and cast levels, respectively in SLE patients with sole LN. P values < 0.05 represented statistical significance.

In order to clarify the relationship of CCR6^+^ Th cell subsets with skin and renal impairment in new onset SLE patients, we categorized patients into four subgroups. The first group included SLE patients with sole renal impairment (SK, n = 13), while the second group consisted of SLE patients with skin involvement (SS, n = 20). The third group of SLE patients had both renal and skin impairment (KS, n = 18), and SLE patients with no kidney and skin involvement (NKS, n = 16) made up the fourth group. Our results indicated that patients from SK, SS and KS groups had significantly higher percentages of CCR6^+^ T and Th22 cell populations in comparison to NKS group or HCs (Fig. 5A,B). Similarly, Th17 cells were also elevated in SK and KS groups. However, the SS group did not show any significant changes in comparison to NKS and HC groups (Fig. 5C). In addition, other CCR6^+^ Th cells, like Th17.1 and Th9 did not show any significant changes (data not shown). Moreover, CCR6^−^ Th cells, Th1 (Fig. 5D) and Th2 (data not shown) also did not reveal any significant differences among these different group of patients.

Th22 and Th17 cell populations correlated with clinical parameters in patients with sole LN or lupus skin disease.

As Th22 and Th17 cells were elevated in SLE patients with skin and renal impairment, we also analyzed their correlation with clinical parameters. The percentage of Th22 cells showed a positive correlation with revised cutaneous lupus disease area index (DAI) (P = 0.0046, r = 0.6068; Fig. 5E) and severity index (SI) (P = 0.0010, r = 0.5617 Fig. 5F). Consistent with this, we also observed a positive correlation of Th22 cells with IgG plasma levels (P = 0.0203, r = 0.5143; Fig. 5G), and an inverse correlation with C3 levels (P = 0.0245, r = 0.5008; Fig. 5H), specifically in patients with sole lupus skin disease. Interestingly, SLE patients with sole renal involvement showed a positive correlation of Th22 cells with ESR plasma levels (P = 0.0006, r = 0.8029; Fig. 5I), and Th17 cells with urinary protein levels (P <0.0001, r = 0.8956; Fig. 5J) and casts (P = 0.0214, r = 0.6287 Fig. 5K).

### SLE patients with skin and renal impairment showed an increased concentration of plasma IL-22 levels

**Figure 6:**
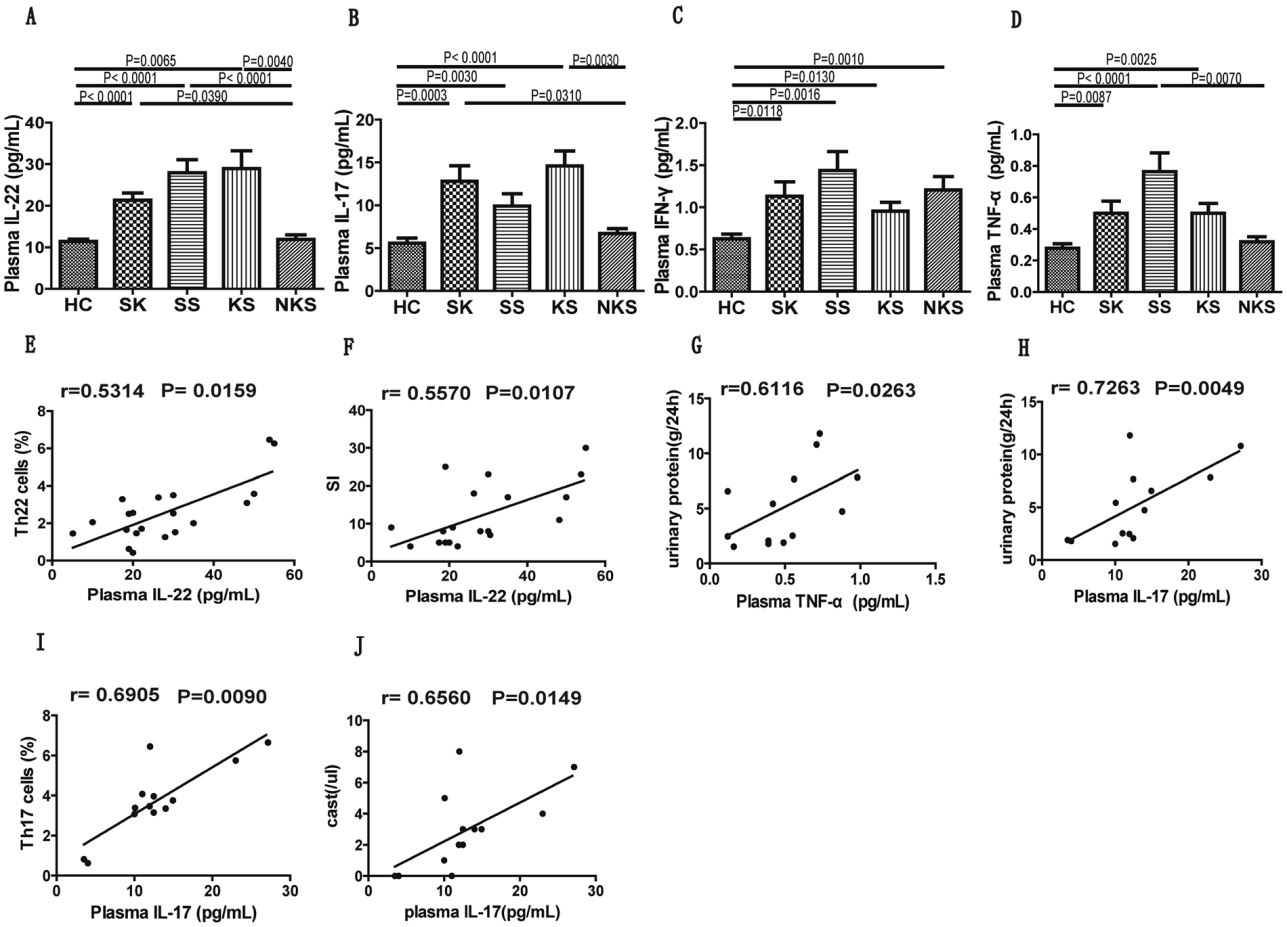
Comparison of plasma levels of cytokines in SLE patients categorized based on organ impairment, and correlation analysis of cytokines plasma levels with Th cell subsets and different clinical parameters of SLE patients with either sole lupus skin disease or sole LN. The plasma levels of IL-22 (panel **A**), IL-17 (panel **B**), IFN-γ (panel **C**) and TNF-α (panel **D**) were analyzed in healthy control and SLE patients from different groups. Differences between groups were analyzed using Kruskal-Wallis ANOVA followed by Dunn-Bonferroni post hoc method. The plasma levels of cytokines IL-22, TNF-α and IL-17 were analyzed for their correlation with Th22, Th17 cell subsets and other clinical parameters, using Spearman’s rank correlation test. Panels (**E**,**F**) represented correlation of plasma IL-22 levels with Th22 cells and SI (severity index) in SLE patients with sole lupus skin disease (n = 20). Panel (**G**) represented plasma TNF-α correlation with urinary protein in SLE patients with sole LN (n = 13). Panel (**H**–**J**) represented plasma IL-17 correlation with urinary protein, Th17 cells and cast, respectively in SLE patients with sole LN. P values < 0.05 represented statistical significance.

As we have observed varying levels of cytokines (IL-22, IL-17, IFN-γ and TNF-α) in SLE patients, we assessed their expression in patient’s specifically categorized based on skin and renal impairment. Our results revealed that concentrations of plasma IL-22 were higher in patients from SK, SS and KS groups, in comparison to NKS and HC groups (Fig. 6A). Similarly, plasma IL-17 level was also significantly elevated in SK and KS group patients compared with NKS and HC group (Fig. 6B). In addition, IL-17 levels in the SS group patients were significantly elevated in comparison to HC group, but not the NKS group. Interestingly, plasma IFN-γ levels were significantly elevated in patients from all groups (SK, SS, KS & NKS) in comparison to HC group (Fig. 6C). This result indicated that changes in IFN-γ levels were not correlated with skin and renal impairment in SLE patients. Finally, patients from SS group showed maximum elevation of plasma TNF-α level, in comparison to NKS and NC group patients. Patients from SK and KS groups showed increased plasma TNF-α levels, but only compared to HC group patients (Fig. 6D).

### Plasma IL-22 levels correlated with the frequency of CCR6^+^ Th22 cells and revised cutaneous lupus disease severity index in SLE patients with sole lupus skin disease

Further correlation analysis of cytokines in SLE patients with sole lupus skin disease revealed that plasma IL-22 levels correlated positively with the percentage of CCR6^+^ Th22 cell population (P = 0.0159, r = 0.5314; Fig. 6E), and the SI (P = 0.0107, r = 0.5570; Fig. 6F). Levels of plasma TNF-α (P = 0.0263, r = 0.6116; Fig. 6G) and IL-17 (P = 0.0049, r = 0.7263; Fig. 6H) positively correlated with levels of urinary protein. In addition, we also observed a positive correlation between plasma IL-17 levels and frequency of Th17 cells (P = 0.0009, r = 0.6905; Fig. 6I) and casts (P = 0.0149, r = 0.6560; Fig. 6J), in patients with sole renal impairment.

## Discussion

In this study, we identified that SLE patients have a higher frequency of circulating CCR6^+^ Th cell subsets, including Th22, Th17 and Th17.1. In addition, SLE patients also showed a higher percentage of CCR6^−^ Th2 cells, but revealed no difference in Th1 population, in comparison to healthy control subjects. These subsets showed a positive correlation with SLEDAI score, and, in addition, showed correlation with other clinical parameters such as urinary protein levels, ESR and C4 protein, in SLE patients. These observations suggest that CCR6^+^ Th cell subsets may play an important role in SLE pathogenesis. Consistent with our findings, previous studies have also established that SLE patients display elevated levels of CCR6^+^ Th22, Th17 cells and CCR6^−^Th2 cells^27–29^. However, to the best of our knowledge, our study is the first to report elevated numbers of CCR6^+^ Th17.1 cells in SLE patients and this subset specifically correlated positively with ESR value, and negatively with C4 levels. The Th17.1 subpopulation is also termed as non-classic Th1 and their respective ontogeny is unclear^30^. This subpopulation may generate during Th17 lymphocyte transition under inflammatory conditions or derive from naive T cells directly in humans^31^, and seems to be particularly aggressive and more pathogenic^30^, especially when there is no response to glucocorticoids, due to expression of multi-drug resistance type 1 (MDR1) transporter gene^32^. This led us to hypothesize that Th17.1 cells may not only play a role in SLE pathogenesis, but also serve as a potential biomarker for the assessment of disease severity.

It has been shown previously that expression of CCR6 on T effector cells positively correlates with the severity of organ impairment in SLE patients^33^, and CCR6/CCL20 axis aids in recruitment of IL-17 producing cells into targeted tissue, and, thus, results in tissue injury^24,33^. To validate these findings and have further clarify the mechanism of CCR6^+^ Th cells in the SLE organ impairment, we stratified SLE patients in our study into different subgroups (SK, SS, KS and NSK) based on skin and renal impairment. The analysis of CCR6 expression on T cells in these different groups indicated that patients from SK, SS and KS group had higher frequencies of CCR6^+^ T cells compared with NKS group. This observation suggested that CCR6^+^ T cells might play pivotal role in skin and renal impairment during SLE.

Th22 cells express multiple skin homing receptors, including CCR6, CCR4 and CCR10^16,18^, which allow these cells to infiltrate into the skin, and thus highlighting the importance of this T cell subset in skin impairment. It has also been shown that Th22 cells and IL-22 overexpression are not only associated with psoriasis but also with various skin diseases, such as atopic dermatitis and scleroderma^34^. Consistent with these published reports, our data also indicated that SLE patients with skin only impairment (SS group) had elevated CCR6^+^ Th22 cells in comparison to NSK group. Also, the Th22 cells frequency in these patients positively correlated with disease area, severity of skin lesions, and IgG plasma concentrations, while negatively correlated with C3 protein levels. This co-relational analysis emphasized that CCR6^+^ Th22 cells might play a pertinent role in the pathogenesis of lupus involving skin issues. Furthermore, as IL-22 is the main effector cytokine of Th22 cells, we also observed elevated levels of plasma Il-22 in SS group patients. There existed a positive correlation between elevated level of plasma IL-22 and CCR6^+^ Th22 cells and SI value. Typically, IL-22 has been shown to be involved in inducing keratinocyte proliferation and causing epidermis hyperproliferation, resulting in premature keratinocytes^35^. The rate of keratinocyte apoptosis has also been noticed to be higher in lesional epidermis of cutaneous lupus erythematosus (CLE) patients, and the degree of apoptosis was same between SLE and CLE patients^36^. In this regard, TNF-α, also secreted by Th22 cells^15,16,37^, has been linked with improving keratinocyte responsiveness to IL-22 by upregulating the expression of IL-22 receptor along with additional signal transduction elements^38^. Interestingly, in our study, we also observed that plasma TNF-α level was increased in SS group patients, thereby suggesting that TNF-α may interact with IL-22 to play a critical role in the pathogenesis of lupus skin impairment. Based on these observations, we speculated that the possible mechanism of Th22 cell involvement in lupus skin disease is that an increased number of CCR6^+^ Th22 cells migrate into the inflammatory location in the skin lesion, and the chemokine receptors expressed by these cells interact with their ligands to produce specific cytokines, which, inturn, induce keratinocyte proliferation. However, to maintain a dynamic balance between apoptosis and proliferation, more keratinocytes undergo apoptosis, but due to impaired or delayed removal of apoptotic cells in SLE^39^, accumulated apoptotic keratinocytes undergoes secondary necrosis, and eventually cause local tissue inflammation. This may trigger a positive feedback loop, resulting in persistent inflammation.

In SLE, renal impairment is one of the other most severe and devastating manifestations. Thus, in our study, we compared the SLE patients with various renal impairment (SK, and NSK group patients). Interestingly, we observed a positive correlation between elevated Th22 levels and ESR in SK group patients. Since the patients in our study were in early stages of the disease, we proposed that Th22 cells might also play a role in the pathogenesis of LN in patients with early onset SLE. In SK group patients, we identified elevated plasma levels of IL-22 in comparison to patients in the NSK group. It has been noted that kidney cells express IL-22 receptor (IL-22R) and blockade of IL-22 results in alleviation of renal injury and improved the clearance of the pathogens in polymicrobial peritonitis. This indicated that IL-22 is detrimental for kidney regeneration^40^. Also, it has been shown that IL-22 could induce complement proteins^41^, and thus has the ability to mediate disease by indirectly affecting the glomerular endothelium in LN through complement. Moreover, TNF-α has also been observed to be elevated, and shows correlation with urinal protein level in SK group patients when compared to HC group. There have also been other studies that implicated TNF-α as an important pathogenic cytokine in LN, and it also contributed to the subsequent LN development^42,43^.

Another T cell subset, which showed higher frequency in patients from SK group, was Th17 cells. Elevated Th17 cell percentage positively correlated with both levels of urinary protein and cast in these patients. Furthermore, we also observed a significant increase in plasma IL-17 levels in SK group patients, which again showed a positive correlation with the percentage of Th17 cells, urinary protein level and casts. Overall, our results were consistent with previously published similar studies^44,45^ and points to the notion that Th17/IL-17 axis might also be contribute to the pathogenesis of LN.

Surprisingly, we did not observe any significant difference in the levels of circulating Th1 cells between SLE patients and HCs, even though SLE patients had significantly higher levels of IFN-γ. This discrepancy may be accounted for due to; 1) Th1 cells may not be the major source of plasma IFN-γ, and this notion is supported by the fact that we did not observe any significant correlation between the plasma IFN-γ level and the percentage of Th1 cells. Thus, Other IFN-γ-producing cells, such as γδT cells, may be responsible for the elevated IFN-γ level in SLE. 2) We also observed an elevated percentage of Th17.1 in SLE patients, and it is highly possible that these cells might be contributing to the elevation of the IFN-γ level and the pathogenesis of SLE.

To summarize, our findings suggested that CCR6^+^ Th22 cells may play an important role in skin or renal impairment in SLE patients, and further proposed CCR6 as a probable therapeutic target for SLE treatment. It is important to mention that our study only detected CCR6^+^ Th cell subsets in the patients’ blood, instead of identifying their infiltration to the target tissues. Furthermore, our study only focused on CCR6^+^ Th cell subsets and their relation with SLE pathogenesis, despite the evidence about the role of other Th cell subsets also. In addition, further studies about the mechanistic role of CCR6^+^ Th cells, especially the CCR6^+^ Th22 cell population on parenchymal cells, and their interaction in the tissues of target organs, would be warranted. In our future studies, we would also like to investigate the involvement of other T cells subsets, including γδT, Tfh and T_reg_ cells in organs impairment including neurological system in SLE, by using larger sample size and multiple time points.

## Methods

### Patients and controls

Our study included a total of 67 new-onset SLE patients with clinical symptoms for less than three months and without any treatment. They were recruited between January and November 2016, from the in-patient department of the First Hospital of Jilin University, China. In addition, we also enrolled 26 healthy volunteers, as controls, matched for ethnicity, age, and gender, from the Physical Examination Center of the outpatient department in our hospital. SLE patients were diagnosed based on the revised criteria for the classification of SLE, by the American College of Rheumatology (ACR)^46^. SLEDAI was applied to assess the disease activity, and an SLEADI score of >6 represented active disease^47^. Moreover, based on ACR criteria, complicated LN was diagnosed if the patient displayed any one of the following signs: (i) persistent urinary protein (≥0.5 g/24 h); (ii) indication of active cellular casts; and (iii) positive biopsy of LN^48^. Also, the shape of a rash and/or the histopathology of skin lesions indicated complicated lupus skin disease. Importantly, subjects with systemic sclerosis, myositis, other autoimmune disorders, cancer, recent infection or who had received glucocorticoid or immunosuppressive treatment within the past six months, were excluded from the study. All the individual subjects gave the written informed consent and experimental protocol was established according to the guidelines of the Declaration of Helsinki and approved by the Human Ethics Committee of Jilin University.

### Clinical examination

The demographic and clinical characteristics of the subjects before treatment, including age and gender were obtained from hospital records. The routine laboratory tests, including determination of complete blood cell counts, concentrations of plasma C3, C4, ESR, CRP, IgG levels, anti-dsDNA and anti- Sm antibodies were measured as described previously^27^. Disease Area and Severity Index (RCLASI: DAI and SI) were calculated in the patients with lupus skin disease, and the urinary protein levels were determined in LN patients for 24 hour time periods.

### Isolation of peripheral blood mononuclear cells (PBMCs)

Peripheral blood (6 mL) was collected from each participant at baseline after an overnight fasting, and PBMCs were isolated by density-gradient centrifugation at 800 g for 30 min using Ficoll-Paque Plus method (Amersham Biosciences, Buckinghamshire, UK). The isolated cells were then resuspended at a concentration of 1 × 10^6^ per mL in RPMI-1640 culture medium (Invitrogen, Carlsbad, CA, USA) containing 10% fetal calf plasma.

### Flow cytometry analysis

The isolated PBMCs were stained in duplicate with APC-H7-anti-CD4 (Becton Dickinson, San Diego, CA, USA), PerCP-Cy5.5-anti-CD3 (Becton Dickinson), BV510-anti-CCR4 (Becton Dickinson), PE-Cy7-anti-CCR6 (Becton Dickinson), BB515-anti-CCR10 (Becton Dickinson), and PE-CF594-anti-CXCR3 (Becton Dickinson) antibodies in the dark at 4 °C for 30 min. Negative controls were stained with isotype-matched control antibodies (APC-H7-anti-IgG1, PerCP-Cy5.5-anti-IgG1, BV510-anti-IgG1, PE-Cy7-anti-IgG1, BB515-anti-IgG2a, and PE-CF594-anti-IgG1). The frequencies of different T cells subsets were determined by flow cytometry analysis using FACSAria II (Beckton-Dickinson, San Diego, CA, USA) and analyzed by FlowJo software from Microsoft (v7.6.2, TreeStar, San Carlos, CA).

### Detection of plasma IL-22 by enzyme-linked immunosorbent assay (ELISA)

The concentration of plasma IL-22 in each participant was determined by ELISA using a specific cytokine kit, according to the manufacturer’s protocol (Affymetrix eBioscience). The levels were calculated according to the standard curve established using recombinant human IL-22 provided in the kit, which had a detection limit of 5 pg/mL.

### Cytometric bead array (CBA) analysis of plasma cytokines

The plasma levels of IL-17A, TNF-α and IFN-γ cytokines were determined using CBA method^49^, according to the manufacturer’s instructions (BD Biosciences) with minor modifications. The quantification was performed using Cell Quest Pro software according to the routine procedures and CBA software (Becton Dickinson) on a FACSAria II.

### Statistical analysis

All statistical analyses were performed using SPSS 21.0 (SPSS, Chicago, IL, USA) software. Quantitative data were represented as individual values or median (range) of each group. Differences between groups were analyzed using Kruskal-Wallis ANOVA followed by Dunn-Bonferroni post hoc method or a Mann-Whitney U test, when appropriate. Correlation analysis was performed using Spearman’s rank correlation test. A two-sided P value of < 0.05 represented statistical significance.

## References

[1] Kaul A (2016). Systemic lupus erythematosus. *Nature reviews*. Disease primers.

[2] Herrmann M, Voll RE, Kalden JR (2000). Etiopathogenesis of systemic lupus erythematosus. Immunology today.

[3] Robak E (2001). Lymphocyctes Tgammadelta in clinically normal skin and peripheral blood of patients with systemic lupus erythematosus and their correlation with disease activity. Mediators of inflammation.

[4] Enghard P (2009). CXCR3 + CD4 + T cells are enriched in inflamed kidneys and urine and provide a new biomarker for acute nephritis flares in systemic lupus erythematosus patients. Arthritis and rheumatism.

[5] Crispin JC (2008). Expanded double negative T cells in patients with systemic lupus erythematosus produce IL-17 and infiltrate the kidneys. Journal of immunology.

[6] Baekkevold ES (2005). A role for CCR4 in development of mature circulating cutaneous T helper memory cell populations. The Journal of experimental medicine.

[7] Annunziato F (2007). Phenotypic and functional features of human Th17 cells. The Journal of experimental medicine.

[8] Kohler RE, Caon AC, Willenborg DO, Clark-Lewis I, McColl SR (2003). A role for macrophage inflammatory protein-3 alpha/CC chemokine ligand 20 in immune priming during T cell-mediated inflammation of the central nervous system. Journal of immunology.

[9] Ruth JH (2003). Role of macrophage inflammatory protein-3alpha and its ligand CCR6 in rheumatoid arthritis. Laboratory investigation; a journal of technical methods and pathology.

[10] Schaerli P (2004). Characterization of human T cells that regulate neutrophilic skin inflammation. Journal of immunology.

[11] Bonecchi R (1998). Differential expression of chemokine receptors and chemotactic responsiveness of type 1 T helper cells (Th1s) and Th2s. The Journal of experimental medicine.

[12] Homey B (2002). CCL27-CCR10 interactions regulate T cell-mediated skin inflammation. Nature medicine.

[13] Sallusto F, Mackay CR, Lanzavecchia A (2000). The role of chemokine receptors in primary, effector, and memory immune responses. Annual review of immunology.

[14] Iwakura Y, Ishigame H, Saijo S, Nakae S (2011). Functional specialization of interleukin-17 family members. Immunity.

[15] Trifari S, Kaplan CD, Tran EH, Crellin NK, Spits H (2009). Identification of a human helper T cell population that has abundant production of interleukin 22 and is distinct from T(H)−17, T(H)1 and T(H)2 cells. Nature immunology.

[16] Duhen T, Geiger R, Jarrossay D, Lanzavecchia A, Sallusto F (2009). Production of interleukin 22 but not interleukin 17 by a subset of human skin-homing memory T cells. Nature immunology.

[17] van Hamburg JP (2013). IL-17/Th17 mediated synovial inflammation is IL-22 independent. Annals of the rheumatic diseases.

[18] Mahnke YD, Beddall MH, Roederer M (2013). OMIP-017: human CD4( + ) helper T-cell subsets including follicular helper cells. Cytometry. Part A: the journal of the International Society for Analytical Cytology.

[19] Acosta-Rodriguez EV (2007). Surface phenotype and antigenic specificity of human interleukin 17-producing T helper memory cells. Nature immunology.

[20] Ye ZJ (2012). Differentiation and recruitment of Th9 cells stimulated by pleural mesothelial cells in human Mycobacterium tuberculosis infection. PloS one.

[21] Rivino L (2004). Chemokine receptor expression identifies Pre-T helper (Th)1, Pre-Th2, and nonpolarized cells among human CD4 + central memory T cells. The Journal of experimental medicine.

[22] Paulissen SM (2015). CCR6( + ) Th cell populations distinguish ACPA positive from ACPA negative rheumatoid arthritis. Arthritis research & therapy.

[23] Paulissen SM (2015). van Hamburg, J. P., Dankers, W. & Lubberts, E. The role and modulation of CCR6 + Th17 cell populations in rheumatoid arthritis. Cytokine.

[24] Turner JE (2010). CCR6 recruits regulatory T cells and Th17 cells to the kidney in glomerulonephritis. Journal of the American Society of Nephrology: JASN.

[25] Hirota K (2007). Preferential recruitment of CCR6^−^expressing Th17 cells to inflamed joints via CCL20 in rheumatoid arthritis and its animal model. The Journal of experimental medicine.

[26] Zhou XJ (2015). *Association of variants in* CCR6 with susceptibility to lupus nephritis in Chinese. Arthritis & rheumatology.

[27] Zhao L (2013). IL-22 + CD4 + T-cells in patients with active systemic lupus erythematosus. Experimental biology and medicine.

[28] Qin WZ (2011). Expressions of IL-22 in circulating CD4 + /CD8 + T cells and their correlation with disease activity in SLE patients. Clinical and experimental medicine.

[29] Amel-Kashipaz MR (2001). Quantitative and qualitative analysis of the balance between type 1 and type 2 cytokine-producing CD8(−) and CD8(+) T cells in systemic lupus erythematosus. Journal of autoimmunity.

[30] Cosmi L, Liotta F, Maggi E, Romagnani S, Annunziato F (2014). Th17 and non-classic Th1 cells in chronic inflammatory disorders: two sides of the same coin. International archives of allergy and immunology.

[31] Zielinski CE (2012). Pathogen-induced human TH17 cells produce IFN-gamma or IL-10 and are regulated by IL-1beta. Nature.

[32] Ramesh R (2014). Pro-inflammatory human Th17 cells selectively express P-glycoprotein and are refractory to glucocorticoids. The Journal of experimental medicine.

[33] Koga T (2016). *Calcium/Calmodulin-Dependen*t Kinase IV Facilitates the Recruitment of Interleukin-17-Producing Cells to Target Organs Through the CCR6/CCL20 Axis in Th17 Cell-Driven Inflammatory Diseases. Arthritis & rheumatology.

[34] Fujita H (2013). The role of IL-22 and Th22 cells in human skin diseases. Journal of dermatological science.

[35] Dudakov JA, Hanash AM, van den Brink MR (2015). Interleukin-22: immunobiology and pathology. Annual review of immunology.

[36] Skiljevic D, Bonaci-Nikolic B, Brasanac D, Nikolic M (2016). Apoptosis of keratinocytes and serum DNase I activity in patients with cutaneous lupus erythematosus: relationship with clinical and immunoserological parameters. Journal of the European Academy of Dermatology and Venereology: JEADV.

[37] Eyerich S (2009). Th22 cells represent a distinct human T cell subset involved in epidermal immunity and remodeling. The Journal of clinical investigation.

[38] Wolk K (2009). IL-22 and IL-20 are key mediators of the epidermal alterations in psoriasis while IL-17 and IFN-gamma are not. Journal of molecular medicine.

[39] Ren Y (2003). Increased apoptotic neutrophils and macrophages and impaired macrophage phagocytic clearance of apoptotic neutrophils in systemic lupus erythematosus. Arthritis and rheumatism.

[40] Weber GF (2007). Inhibition of interleukin-22 attenuates bacterial load and organ failure during acute polymicrobial sepsis. Infection and immunity.

[41] Hasegawa M (2014). Interleukin-22 regulates the complement system to promote resistance against pathobionts after pathogen-induced intestinal damage. Immunity.

[42] Imaizumi T (2015). Tumor necrosis factor-alpha synergistically enhances polyinosinic-polycytidylic acid-induced toll-like receptor 3 signaling in cultured normal human mesangial cells: possible involvement in the pathogenesis of lupus nephritis. Clinical and experimental nephrology.

[43] Aringer M (2009). Adverse events and efficacy of TNF-alpha blockade with infliximab in patients with systemic lupus erythematosus: long-term follow-up of 13 patients. Rheumatology.

[44] Zickert A (2015). IL-17 and IL-23 in lupus nephritis - association to histopathology and response to treatment. BMC immunology.

[45] Kwan BC (2009). The gene expression of type 17 T-helper cell-related cytokines in the urinary sediment of patients with systemic lupus erythematosus. Rheumatology.

[46] Tan EM (1982). The1982 revised criteria for the classification of systemic lupus erythematosus. Arthritis and rheumatism.

[47] Bombardier C, Gladman DD, Urowitz MB, Caron D, Chang CH (1992). Derivation of the SLEDAI. A disease activity index for lupus patients. The Committee on Prognosis Studies in SLE. Arthritis and rheumatism.

[48] Schiffenbauer J, Simon LS (2004). Randomized controlled trials in systemic lupus erythematosus: what has been done and what do we need to do?. Lupus.

[49] Morgan E (2004). Cytometric bead array: a multiplexed assay platform with applications in various areas of biology. Clinical immunology.

